# Design and Experimental Evolution of *trans*-Splicing Group I Intron Ribozymes

**DOI:** 10.3390/molecules22010075

**Published:** 2017-01-02

**Authors:** Ulrich F. Müller

**Affiliations:** Department of Chemistry & Biochemistry, University of California, San Diego, CA 92093-0356, USA; ufmuller@ucsd.edu; Tel.: +1-858-534-6823

**Keywords:** ribozyme, evolution, splicing

## Abstract

Group I intron ribozymes occur naturally as *cis*-splicing ribozymes, in the form of introns that do not require the spliceosome for their removal. Instead, they catalyze two consecutive *trans*-phosphorylation reactions to remove themselves from a primary transcript, and join the two flanking exons. Designed, *trans*-splicing variants of these ribozymes replace the 3′-portion of a substrate with the ribozyme’s 3′-exon, replace the 5′-portion with the ribozyme’s 5′-exon, or insert/remove an internal sequence of the substrate. Two of these designs have been evolved experimentally in cells, leading to variants of group I intron ribozymes that splice more efficiently, recruit a cellular protein to modify the substrate’s gene expression, or elucidate evolutionary pathways of ribozymes in cells. Some of the artificial, *trans*-splicing ribozymes are promising as tools in therapy, and as model systems for RNA evolution in cells. This review provides an overview of the different types of *trans*-splicing group I intron ribozymes that have been generated, and the experimental evolution systems that have been used to improve them.

## 1. Introduction

Self-splicing group I introns represent one of the two first known catalytic RNAs (ribozymes) [1,2]. Their ability to perform self-splicing means that these introns do not require the spliceosome to be removed from the primary transcript: They catalyze two transphosphorylation reactions to excise themselves and join the two flanking exons. In some cases, the genomic sequence encoding these ribozymes is separated into several fragments such that the ribozyme needs to assemble from two different transcripts [3].

This review focuses on different, artificial designs of *trans*-splicing ribozymes, in which the substrate resides on a different strand than the ribozyme. The earliest design of a *trans*-splicing group I intron ribozyme was used to aid the in vitro analysis of group I intron reactivity [4], using short RNA oligonucleotides as substrate to simplify the analysis. Later, the interest shifted to long RNA substrates such as mRNAs, mediating the sequence modification of naturally occurring mRNAs in vitro and in cells [5]. Additional designs of substrate recognition for these ribozymes led to a total of five types of interactions between *trans*-splicing group I intron ribozymes and cellular RNAs [5,6,7,8]. This review summarizes the design and analysis of different *trans*-splicing group I intron ribozymes, and their artificial evolution for possible use as an evolutionary model system in RNA biochemistry, or as a tool in therapeutic applications.

## 2. Group I Intron Ribozymes

**Figure 1:**
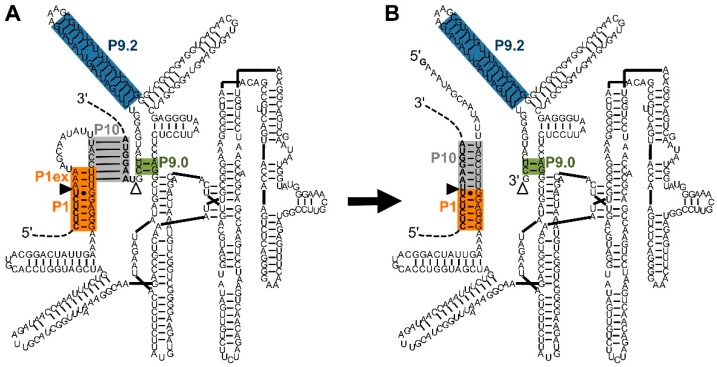
Secondary structure and reaction of the natural, *cis*-splicing group I intron ribozyme from *Tetrahymena*. The shown secondary structure is based on the structure by Cech et al. [24] with the exception that the P4-P6 domain was placed on the right to clarify interactions at the 5′-splice site and 3′-splice site. Additionally, the P10 helix (grey) is shown in the same structure (**A**) as the P1 helix extension (orange). The display of both helices helps envision the conformational change between the two catalytic steps although these two helices do not exist at the same time. The intron is the sequence between the 5′-splice site (filled triangle) and the 3′-splice site (empty triangle). The P1 helix (orange) and P1 helix extension (P1ex; orange) define the 5′-splice site at the G:U pair. The P10 helix (grey), and P9.0 helix (green), with help from the P9.2 helix (blue), define the 3′-splice site. During splicing (from (**A**) to (**B**)), the P1 helix extension is opened to reveal the 3′-hydroxyl group of the terminal uridine at the 5′-splice site. The P10 helix then mediates a conformational change, in which the 3′-exon (upper dashed line) is positioned adjacent to the 5′-exon (lower dashed line), allowing the nucleophilic attack of the 3′-uridine the 3′-splice site, joining 5′-exon and 3′-exon.

Since their discovery in 1982, group I intron ribozymes have transformed our understanding of RNA structure and function because they were widely used as objects to study RNA structure [9,10,11,12,13], RNA folding [14,15,16,17,18], and RNA catalysis [19,20,21,22]. Their reaction pathway includes one catalytic step, a conformational change, and a second catalytic step. In the first catalytic step, the 3′-hydroxyl of an exogenous guanosine attacks the 5′-splice site and separates the 5′-exon from the intron [23]. Via a conformational change, which can be aided by P10 helix formation (Figure 1), the terminal guanosine of the intron enters the active site. In the second catalytic step, the 3′-terminal uridine of the 5′-exon attacks the phosphordiester bond at the 3′-splice site [22], joining the 5′-exon and 3′-exon (Figure 1B) [1].

The group I intron ribozyme from *Tetrahymena* is relatively robust to different sequence contexts and reaction conditions [5,25,26]. The group I introns from *Pneumocystis carinii* [27], *Candida albicans* [28], and the myxomycetes *Didymium* and *Fuligo* [25] have shown similar potential, while the well-studied group I intron from *Azoarcus* is not suitable as a versatile *trans*-splicing ribozyme due to very specific substrate secondary structure requirements [29]. The majority of studies on *trans*-splicing group I intron ribozymes focused on the ribozyme from *Tetrahymena*.

## 3. *trans*-Splicing with Group I Intron Ribozymes

**Figure 2:**
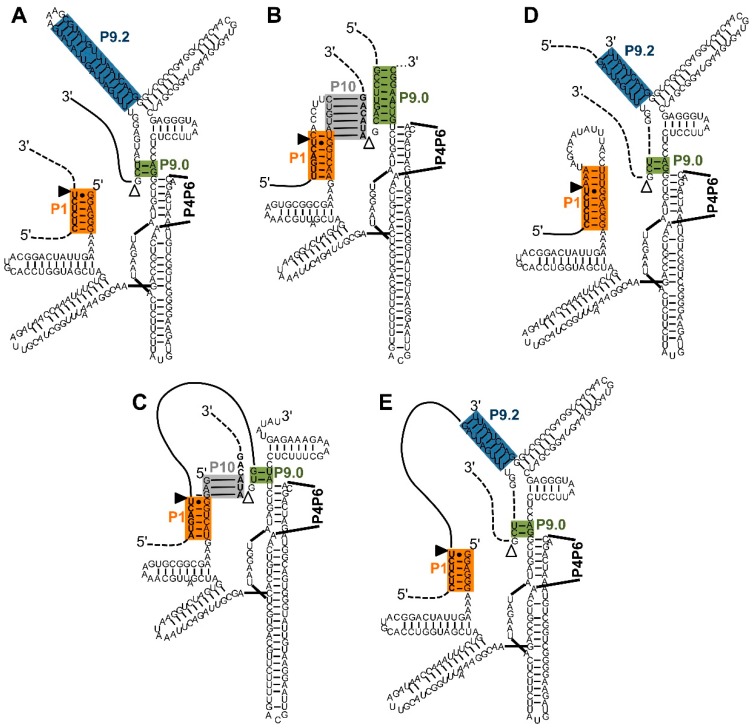
Five types of interactions between *trans*-splicing ribozymes and their substrates. Note that the P4–P6 domains are not shown for clarity. Color-coding and the (non-physical) overlap of helices P1 extension and P10 are as described in Figure 1. (**A**) Secondary structure of a *Tetrahymena* ribozyme variant designed for *trans*-splicing at the 5′-splice site [5]. During two *trans*phosphorylation reactions, the 3′-portion of the substrate (dashed line) is replaced by the 3′-exon of the ribozyme construct (solid line); (**B**) Secondary structure of a *Pneumocystis carinii* ribozyme variant designed for *trans*-splicing at the 3′-splice site [6]. The substrate is positioned to the 3′-splice site by the P10 and P9.0 helices. During two transphosphorylation reactions, the 5′-portion of the substrate (dashed line) is replaced by the 5′-exon of the ribozyme (solid line). Note that the P9.0 helix is artificially elongated; (**C**) Secondary structure of a *Pneumocystis carinii* ribozyme variant designed for *trans*-splicing at the 5′ and 3′-splice site [30]. The design uses the *trans*-contacts shown in (**A**,**B**). During the transphosphorylation reactions, the internal sequence (solid line) of the substrate is removed, joining the two flanking exons (dashed lines); (**D**) Secondary structure of a *Tetrahymena* ribozyme variant designed for *trans*-splicing at the 3′-splice site [8]. The P9.2 and P9.0 helices position the substrate to the 3′-splice site. During the transphosphorylation reactions the 5′-portion of the substrate (dashed line) is replaced by the 5′-exon of the ribozyme construct (solid line); (**E**) Secondary structure of a *Tetrahymena* ribozyme variant designed for *trans*-splicing at the 5′ and 3′-splice site [8]. The design uses the *trans*-contacts shown in (**A**,**D**). During the transphosphorylation reactions, the internal sequence (solid line) of the substrate is removed, and the two flanking exons (dashed lines) are joined.

*trans*-Splicing group I intron ribozymes recognize their target sites on substrate RNAs by base pairing. Five different designs have been used in which the 5′-splice site, the 3′-splice site, or both splice sites are recognized (Figure 2). Three of these designs splice at a single splice site: (i) the 5′-splice site is recognized by formation of the P1 helix; (ii) the 3′ splice site is recognized by a combination of P10 and P9.0 helix formation; and (iii) the 3′-splice site is defined by formation of the P9.2 helix. This results in ribozymes that (i) replace the 3′-portion of the substrate with the 3′-exon of the ribozyme construct; or (ii and iii) replace the 5′-portion portion of the substrate with the 5′-exon of the ribozyme construct. Two additional designs combine splicing at the 5′-splice site with either of the two ways to recognize the 3′-splice site, resulting in ribozymes that insert or delete internal sequences of a substrate RNA. These five constructs are described below, and illustrated in Figure 2A–E.

The first type of *trans*-splicing to be established was *trans*-splicing at the 5′-splice site, where the contact between ribozyme and substrate is formed by the P1 helix (Figure 2A; [4,31,32]). The P1 helix docks into the active site, allowing catalysis to occur at the 5′-splice site [33]. Complete mRNAs were spliced in vitro and in cells with this substrate recognition principle [5]. The second step of splicing (“exon ligation”) then transferred the ribozyme’s 3′-exon to the 5′-portion of the substrate RNA. If the substrate RNA carries a mutation in its 3′-portion and the ribozyme’s 3′-exon represents the ”healthy” sequence then these ribozymes can mediate the repair of a mutated mRNA [5]. For an excellent review on this class of *trans*-splicing ribozymes, see [34].

One way to recognize the 3′-splice site in trans involves the P10 and P9.0 duplexes using the group I intron from *Pneumocystis carinii* (Figure 2B [6,28,35]; for a detailed review see [36]). In natural *cis*-splicing group I intron ribozymes, the P10 duplex forms between the loop of the P1 duplex and nucleotides 2–8 of the 3′-exon [37]. The P9.0 duplex is formed between nucleotides near the 3′-end of the intron and a sequence close to the central core of the ribozyme, thereby helping to position the 5′-splice site near the catalytic site [38]. Both the P10 duplex and the P9.0 duplex are relatively weak, perhaps because they need to open up during the conformational change between the two catalytic steps of splicing. However, *trans*-splicing ribozymes can benefit from stronger P10 and P9.0 duplexes [39] because both duplexes are formed in trans, linking the ribozyme to its substrate [6]. During this type of *trans*-splicing, the substrate 5′-terminus displaces the 5′-exon of the ribozyme construct in the P1 duplex, leading to an overall replacement of the substrate 5′-portion with the 5′-exon of the ribozyme construct [6].

Combining 3′-splice recognition by the P9.0 and P10 helix with 5′-splice site recognition by the P1 duplex generates two splice sites, leading to (i) the excision of an internal sequence (Figure 2C); or (ii) the insertion of an internal sequence into the substrate RNA [7,40], for review see [36]). The excision of short sequences from a substrate RNA works in vitro [7,39]; the excision of single nucleotides has been shown in vitro and in *E. coli* cells [30]. (i) The *trans*-excision reaction (TES) has possible therapeutic applications because it allows for the excision of single nucleotides inside cells to repair insertion mutations, or other frame shift mutations on the RNA level. Unfortunately, the excision efficiency of longer internal sequences seems to be low in cells; (ii) The insertion of short oligomeric sequences into a target RNA is interesting as tool in research because it allows the insertion of modified RNAs into a target sequence in vitro [40,41]. To set up this *trans*-insertion reaction (TIS), the ribozyme is first ”charged” by letting it catalyze the reverse of the second catalytic step of splicing, linking itself to the short insert sequence containing the modification. The *trans*-insertion reaction itself then represents the reverse of the *trans*-excision reaction. This activity could be useful in areas of RNA biochemistry where the chemical modification of specific positions in a long, otherwise unmodified RNA is desirable: The short, modified RNA could be inserted into the specific site of the long RNA [40], for example to position nucleotides with thiol-modifications for biochemical analysis, or isotopically labelled nucleotides for NMR analysis.

A second way to recognize the 3′-splice site utilizes the P9.0 and P9.2 helices in the *Tetrahymena* ribozyme (Figure 2D) [8]. Here, the P9.2 helix is formed in trans between the ribozyme 3′-terminus and a target sequence on the substrate. The minimal length of this *trans*-P9.2 helix is 6–7 base pairs, similar to the length requirement of the P1 helix. The reaction mechanism is identical to that of the natural *cis*-splicing ribozyme, only that the P9.0 and P9.2 duplex are formed in trans, between ribozyme 3′-terminus and the substrate. This leads to the replacement of the substrate’s 5′-portion with the 5′-exon of the ribozyme.

When this 3′-splice site recognition by the P9.2 duplex is used together with the 5′-splice site recognition by the P1 duplex then introns with a length of 100 nucleotides can be removed from RNA substrates, in vitro and in *E. coli* cells (Figure 2E) [8]. These ribozymes were termed ”spliceozymes” because they act analogously to the spliceosome, removing long intron sequences and joining the flanking exons in cells. This approach has the advantage of higher efficiency and the ability to remove long inserts over the *trans*-excision reaction (see above). However, one important drawback of this design is that four nucleotides in the substrate upstream of the 3′-splice site are confined to a specific sequence. This currently prevents the ribozyme from being used as a general tool. The remaining sequence of the removed intron in the substrate RNA can be quite diverse, with about 3 in 10 arbitrary 100-nucleotide intron sequences resulting in splicing efficiencies in the range of 3% [8].

*trans*-Splicing efficiencies are highly dependent on the expression levels of the ribozymes in cells: The strong bacteriophage T7 RNA polymerase promoter resulted in up to 50% substrate RNAs converted to products, while the medium strength RNA Polymerase II promoter gave up to 9% conversion [42]. Similarly, the ratio of expression level between ribozyme affects the splicing efficiency, where one case with a ratio of 10:1 for ribozyme: substrate gave 25% efficiency, while a ratio of 100:1 resulted in 49% efficiency [43]. While it is challenging to compare splicing efficiencies from different studies inside cells, recognition of the 5′-splice site by the P1 helix [42], typically in the range up to 10% efficiency [34], appears similarly efficient to constructs where in addition, the 3′-splice site is recognized by the P10/P9.0 helices (for the excision of a single nucleotide [30]) and constructs where the 3′-splice site is recognized by the P9.2 helix (for the excision of 100 nucleotides [8]).

Multiple-turnover splicing should, in principle, be possible for both ribozyme designs excising internal sequences from a substrate RNA (TES and spliceozymes) because the sequence of these ribozymes does not differ before and after the reaction [7,8]. However, multiple turnover would require the dissociation of the ribozymes from the splicing products. None of the studied constructs have shown multiple turnover [8,39], suggesting that interactions with the target sites are currently too strong for product dissociation.

## 4. Identification of Efficient Splice Sites on Target RNAs

All uridine residues in a substrate RNA are possible target sites for *trans*-splicing ribozymes because the only strict sequence requirements for splicing at the 5′-splice site are the formation of a P1 helix [31,32] and the presence of a G:U pair, with the uridine on the substrate strand [44,45]. However, most uridine residues within a substrate RNA are not accessible, reducing the number of splice sites to a small fraction [46].

To experimentally identify efficient splice sites on a substrate RNA, a *trans*-tagging assay was developed [47]. Here, a ribozyme library with the 5′-terminal sequence 5′-GNNNNN ... reacts with the substrate, and splice sites are identified by reverse transcription and sequencing of the products. This *trans*-tagging assay was used successfully by many different labs to identify efficient splice sites [25,48,49,50].

To predict efficient splice sites computationally, the binding free energies were calculated by summing the computed energies of unfolding the target site, unfolding of the ribozyme 5′-terminus, and hybridizing target site and ribozyme 5′-terminus [51]. This method gave an excellent correlation between computational prediction and experiment. The computational prediction has advantages over the experimental *trans*-tagging assay because the computational approach avoids artifacts from the preferred transcription of library molecules with purines at the 5′-terminus [52], from biases from the preferential PCR amplification of shorter amplicons [53]) and from the different tendency for self-inactivation by premature cleavage at the 3′-splice site [51]. One caveat with the computational approach to predict efficient splice sites is that, so far, it has only been tested on the *CAT* mRNA. However, each of the 18 tested splice sites is flanked by a different secondary structure, therefore the results on *CAT* mRNA are probably representative of a general strength for the computational prediction of splice sites.

## 5. Identification of Efficient Extended Guide Sequences on the Ribozyme 5′-Termini

**Figure 3:**
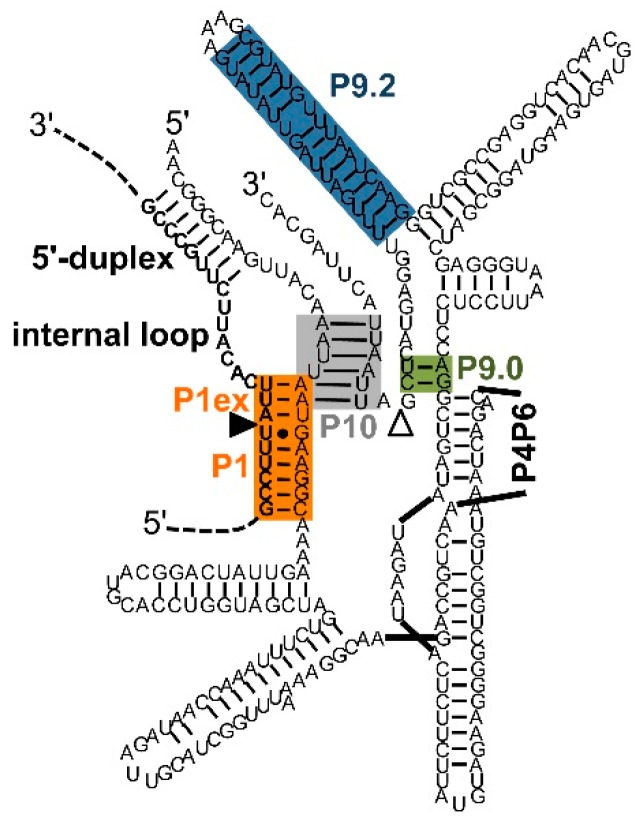
Secondary structure elements formed between extended guide sequence (EGS) and substrate [50,54]. Color-coding and the (non-physical) overlap of helices P1 extension and P10 are as described in Figure 1. The EGS is the ribozyme sequence 5′-terminal of the G at the 5′-splice site. The secondary structure elements formed between EGS and substrate are P1 extension, internal loop, and 5′-duplex. The secondary structure element formed between EGS and the 3′-exon of the ribozyme construct is the P10 helix. The secondary structure is that of a *trans*-splicing *Tetrahymena* ribozyme with an EGS optimized in a selection experiment [50]. The sequence of the 3′-exon was modified to illustrate the P10 helix.

The efficiency of *trans*-splicing can be increased when the ribozyme 5′-terminus is extended further than what is required to form the P1 helix [54]. The additional secondary structure elements formed by this elongation are a P1 extension of ~3 base pairs [50,54,55], an internal loop, and a further duplex between a part of the extended guide sequence (EGS) termed the ”antisense region” [54] and the substrate (Figure 3).

The optimal length of the P1 extension helix past the 5′-splice site is exactly three base pairs. Shorter helices [54] and longer helices [50] mediate less activity. The length of three base pairs corresponds to the length of the natural P1 extension in the *cis*-splicing *Tetrahymena* ribozyme [31].

The optimal length of the terminal EGS duplex (the “antisense region”) is less clear: While the *trans*-splicing efficiency is much higher in the presence than in the absence of a duplex, a duplex with 46 base pairs mediated the same effect as duplexes with 100 and 200 base pairs [54]. In a different system, a 35-base pair duplex was sufficient for optimal activity in mammalian cells [42], and an 8-base pair duplex appeared to be sufficiently long in a different context [50]. It is possible that longer antisense regions are helpful for less efficient splice sites because the rate limiting step for annealing lies in the formation of the first three base pairs at any position in the sequence [56], allowing less accessible splice sites to become accessible if the antisense region initiates annealing on an accessible site of the substrate. Applications in mammalian cells may want to avoid longer RNA duplexes because these trigger cellular defense responses such as the interferon-induced double-stranded RNA-dependent protein kinase response [57].

The P10 helix increases the 3′-splice site specificity [58] and may have an optimal length around six base pairs [54]. While a similar length was confirmed by others [42,59] the optimal length appears to differ between splice sites because shorter P10 duplexes can also allow efficient *trans*-splicing in cells [59].

The internal loop formed between EGS and substrate appears to function well when the predicted secondary structure of the loop forms a single-strand of 5–6 nucleotides in the substrate side or in the product [50]. However, the optimal sequence of these single-stranded regions has not been identified [50]: When 12 EGS variants were designed based on the principles described above, and tested for their ability to repair *CAT* mRNA a large difference between the 12 constructs was observed in mediating cellular resistance to chloramphenicol [59]. Therefore, the current best practice may be to design ~10 constructs and experimentally determine the most efficient sequence.

## 6. Selection Systems for Improved *trans*-Splicing Group I Intron Ribozymes

To develop improved ribozyme variants without individually generating and testing many ribozyme constructs, combinatorial methods are of great help. There are two types of combinatorial methods: Selections and evolutions. In selections, a library of partially randomized sequences is generated, and the most efficient sequences are isolated in one or more selection steps. In an evolution, the starting point is not necessarily a library but can be a single sequence. Using mutagenic PCR, mutations are randomly inserted throughout the sequence, and the most efficient variants of this sequence pool are then selected. The defining characteristic of an evolution is that mutagenesis and selection are applied repeatedly. In this way, the evolving population accumulates mutations that can be tolerated, and successively enriches mutations that increase activity. The iterative character of the evolution allows probing a much larger sequence space by evolution than by selection because each selection is limited to exploring the sequence complexity of the initial library, while evolutions continue to generate, test, and enrich new, beneficial mutations. Hybrids of selections and evolutions often select the best variants from a large, initial starting library, then improve the selected variants by multiple rounds of evolution. Examples for selections and evolutions of *trans*-splicing group I ribozymes will be given in the next paragraphs.

Three different selection steps were developed for *trans*-splicing ribozymes catalyzing the forward splicing reaction [50,60,61]. Although the reverse reaction of the second step of splicing has been used to select variants of group I intron ribozymes [62,63,64,65], the ribozymes evolved by this procedure catalyzed only one of the two catalytic steps efficiently and not the two-step splicing process [64]. To facilitate selection for the forward splicing reaction, the ribozymes and their products need to be compartmentalized. The established three different selection steps used cells as compartments, and relied on three different selectable markers: A transcription factor acting on prototrophy genes, beta-lactamase generating a colorimetric assay, and antibiotic resistance genes. The first selection system was established in *S. cerevisiae*. Here, the ribozyme-mediated repair of a GAL4-derived transcription activator allowed the expression of the HIS3 gene to mediate histidine prototrophy, the ADE2 gene mediating adenine prototrophy, and the lacZ gene to mediate beta-galactosidase activity [60]. This selection system was used to select the best variants from a library containing 13 different *trans*-splicing ribozymes. The second selection system used ribozymes targeted to repair beta-lactamase mRNA in monkey kidney cells (COS-1) [61]. The beta-lactamase activity allowed automated sorting of individual cells. The quantification of fluorescence-positive and -negative cells for different ribozyme constructs showed that the system could be used as a selection step for the selection or evolution of *trans*-splicing group I intron ribozymes.

The third selection system, using antibiotic resistance genes as selection marker, was first established on a *cis*-splicing construct, where the excision of active ribozymes from the pre-mRNA of kanamycin nucleotidyltransferase mRNA mediated kanamycin resistance to *E. coli* cells [55]. The study found that the self-excision became more efficient with mutations that weakened the P1 extension duplex. The first use of antibiotic resistance genes as selection marker for *trans*-splicing ribozymes relied on the action of chloramphenicol acetyl transferase (*CAT*) [50]. The *Tetrahymena* group I intron ribozyme was used to repair a *CAT* mRNA that was inactivated by a frame shift mutation in its 3′-portion, leading to *E. coli* growth on medium containing chloramphenicol. From a library of 9 × 10^6^ ribozymes that differed in their extended guide sequence (EGS), six rounds of selection identified eight EGS sequences with significantly increased activity (see Section 5).

## 7. Evolution of Improved *trans*-Splicing Group I Intron Ribozymes

**Figure 4:**
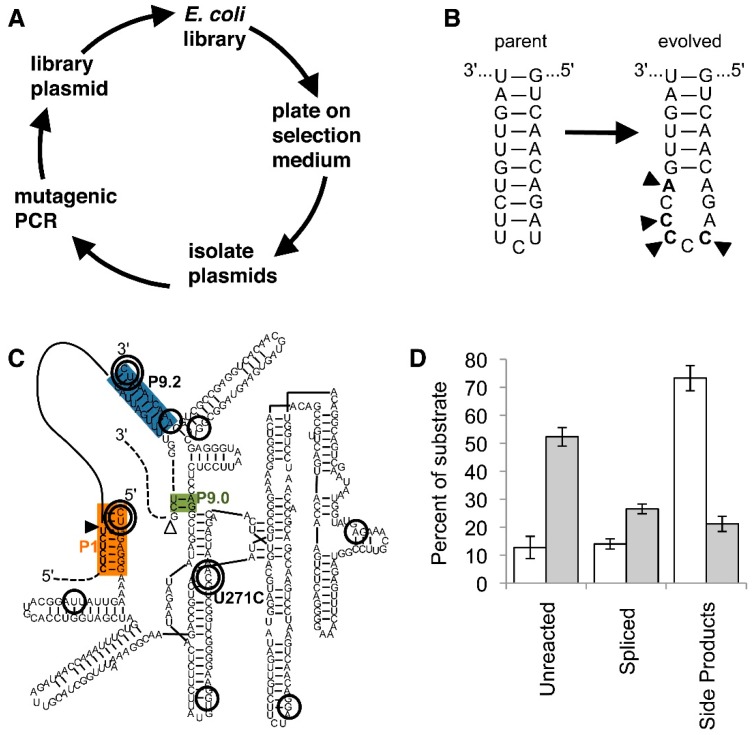
Evolution of *trans*-splicing group I intron ribozymes in cells. Color-coding is as in Figure 1. (**A**) Schematic for one cycle of the evolution procedure. After mutations are introduced into the ribozyme gene by mutagenic PCR the ribozyme gene is cloned into a library plasmid that also expresses the inactivated antibiotic resistance marker. *E. coli* cells transformed with this plasmid only grow on selection medium if the ribozyme is able to repair the antibiotic resistance mRNA. From *E. coli* colonies, plasmids are then isolated, starting a new evolution cycle; (**B**) Secondary structure of the P6b loop in the parent ribozyme (left), and in the evolved ribozyme (right). The four mutations (arrowheads) open the loop and present a (C)_5_ sequence that is specifically bound by the Rho transcription termination factor [66]; (**C**) Secondary structure of an evolved spliceozyme [68]. The 10 mutations of the ”winner” are circled; the most enriched mutations are shown with a double circle. Note the double-circled mutation U271C in the highly-conserved core of the ribozyme; (**D**) In vitro reaction products of the spliceozymes after 60 min. Compared to the parent ribozyme (white columns), the evolved spliceozyme (grey columns) has left four-fold more substrate unreacted, has generated two-fold more correctly spliced product, and has generated one-third of side products. Data are taken from [68].

Three studies describe the evolution of *trans*-splicing ribozymes over multiple rounds of evolution. The first evolution of *trans*-splicing group I intron ribozymes used the *Tetrahymena* ribozyme and proceeded over 21 rounds of evolution [66]. It used the same experimental setup as described in the selection above [50] but repeatedly introduced mutations over the entire length of the ribozyme (Figure 4A). The rate of mutagenesis was varied between 0, 10, 20, and 30 cycles of mutagenic PCR. Mutagenesis levels with 30 cycles of mutagenic PCR (corresponding to ~7.3 mutations per ribozyme) led to a collapse of the evolving population, while 10 or 20 cycles of mutagenic PCR, (~2.4 and 4.8 mutations per ribozyme, respectively) led to stable populations. The effect of recombination [67] was tested with ~1 recombination event per evolution round and ribozyme [66]. The most efficient, evolved motif did not benefit from recombination because it required four clustered mutations. However, recombination appeared to reduce deleterious mutations by at least four-fold. The evolved four mutations in the P6b loop of the ribozyme generated an accessible (C)_5_ homopentamer sequence (Figure 4B), which recruited the Rho transcription factor. Interestingly, the mutations did not increase *trans*-splicing efficiency or transcription efficiency in *E. coli* but strongly benefitted the translation of the spliced mRNA. This evolution illustrates how an evolving population of ribozymes can sample the interaction with cellular components and establish a novel binding site to those proteins that benefit its evolutionary interest.

To test the influence of selection pressure on the success of an evolution the evolution described above was repeated in four parallel branches, two of which operated under the highest selection pressure sustainable, and two of them under low selection pressure [59]. The selection pressure was applied in the form of the chloramphenicol concentration in the selection step. After 12 rounds of different selection pressure, two rounds with high selection pressure were appended to all four branches to enrich for the most active ribozymes. Interestingly, the two branches evolved under low selection pressure had significantly higher pool activity than the two branches evolved under high selection pressure. To identify the cause for this behavior, all 32 evolutionary intermediates of the 5-mutation winning mutant were generated and tested for activity under low, medium, and high selection pressure. The results showed that four of the five mutations acted cooperatively, such that the evolutionary path from wild type to winning mutant could only be walked efficiently under low selection pressure but not under high selection pressure. This behavior was predicted earlier on theoretical grounds [69].

A *trans*-splicing group I intron ribozyme acting on two splice sites, a spliceozyme [8], was evolved in *E. coli* cells recently [68]. Following the lessons learned from previous studies, the most efficient splice site 258 was chosen as target site on *CAT* mRNA [8,51]. An efficient intron with a length of 100 nucleotides was inserted at this splice site to inactivate the *CAT* mRNA, forming a *CAT* pre-mRNA [8]. Short target recognition sites were chosen to allow for the possibility of multiple turnover splicing. The 11-mutation winner of the evolution displayed three classes of mutations necessary and sufficient for the improved activity (Figure 4C): The first class strengthened the target site recognition by extending the complementarity at the 5′-splice site and 3′-splice site by two and one base pairs, respectively. The second class, a single, extremely highly enriched mutation, resided in the highly conserved region of the ribozyme: The U271C mutation appeared to decrease the affinity of the ribozyme for the 5′-splice site, probably by modulating the interaction between the junction 7/8 and the P1 helix. The third class contained six mutations, which at least partially cooperated with the U271C mutation. The effects of the mutations were visible in *E. coli* cells as well as in vitro, demonstrating that no cellular factor was required for their action. Biochemical analysis of all detectable products from the in vitro reaction showed that the overall effect of the mutations was to decrease activity at the 5′-splice site and increase activity at the 3′-splice site, thereby reducing side product formation and increasing product formation (Figure 4D).

## 8. Possible Applications of Evolved *trans*-Splicing Ribozymes

*trans*-Splicing group I intron ribozymes have been explored for two types of applications. First, these ribozymes can repair genetic mutations on the RNA level, by replacing the mutated portion of an mRNA with the 'healthy' sequence [5,48,70]. Second, group I intron ribozymes can selectively kill cells that indicate a disease. For example, cancer cells can be killed by group I introns that target sites on RNAs expressed highly in cancer cells (such as hTert RNA) by splicing a ‘suicide gene’ into the target RNAs. The suicide gene can be the coding region for a cytotoxic peptide such as diphteria toxin A [71], or encode herpes simplex virus thymidine kinase (HSV-tk), which converts the drug ganciclovir to a cytotoxin [72]. The latter approach is currently being tested in clinical trials, demonstrating the real possibility that *trans*-splicing group I intron ribozymes will soon be in therapeutic use [73]. Another example to selectively kill cells indicating a disease targets virally infected cells. Here, *trans*-splicing ribozymes target viral RNAs, which has an advantage in that these targets do not exist in healthy cells. Again, the coding region for cytotoxic peptides or HSV-tk is spliced into the target RNAs, triggering apoptosis of diseased cells [49,71,74]. One of the problems to be overcome is the localized and efficient delivery of the ribozymes into cells [75,76]. Several different approaches are in development, with viral vectors such as the adeno-associated virus being one of the most promising candidates, and modified Salmonella being one of the more recent, creative solutions [77,78].

Group I intron ribozymes could also serve as model systems for the evolution of RNAs in cells. For example, the group II intron shares a common ancestor with the spliceosome [79], and the evolution from a likely ribozyme precursor to a five-partite, multimegadalton, dynamic RNA-protein complex that is able to correctly recognize thousands of splice sites is far from being elucidated. The evolution of group I intron spliceozymes can serve as model for specific biochemical steps in the evolution of the spliceosome: Our previous evolution [68] showed how a ribozyme evolved for *cis*-splicing can adjust its mechanics to the *trans*-reaction, and an evolution with group I introns using a single splice site showed how an evolving RNA can recruit a cellular protein [66]. Further evolution studies may develop variants of group I introns that show more and more characteristics of spliceosomes.
